# Inconsistent Pulse Oximetry Signal in Multiple Sites as an Early Indicator of Severe Hypovolemia

**DOI:** 10.7759/cureus.95085

**Published:** 2025-10-21

**Authors:** Mohamad Taha, Raafay Malik, William T Davis, Sharif Mohamed

**Affiliations:** 1 Anesthesiology, Texas A&M College of Medicine, Bryan, USA; 2 Anesthesiology, University of Houston, Houston, USA; 3 Anesthesiology, University of Texas Medical Branch, Galveston, USA

**Keywords:** anesthesiology, cardiopulmonary resucitation, hypovolemia, pulse oximetry, shock

## Abstract

A low-quality pulse oximetry signal should not be overlooked, as this may indicate cases of severe hypovolemia. Although this sign is not specific for volume depletion, it can provide a quick, non-invasive indication about possible hypovolemia if a proper signal cannot be obtained, especially when difficulties are encountered in obtaining SpO_2_ readings from multiple sites. Severe hypovolemia is a preventable risk factor for shock and other electrolyte abnormalities, which, if untreated, may lead to cardiovascular collapse or even necessitate cardiopulmonary resuscitation (CPR). This case report highlights the potential usefulness of a pulse oximeter's poor signal in multiple sites in helping providers detect volume depletion in the perioperative setting, which could prevent avoidable causes of acute decompensation during anesthetic induction.

## Introduction

Hypovolemia is characterized by a reduction in circulating blood volume that results in decreased venous return, lower cardiac output, and reduced arterial pressures, all of which compromise oxygen transport to peripheral tissues and vital organs [1]. Without proper identification and correction, hypovolemia can progress rapidly to hypovolemic shock, where circulatory failure promotes inadequate end-organ perfusion, leading to potential multiorgan dysfunction and death [2].

Hypovolemic shock remains a significant contributor to morbidity and mortality both inside and outside the operating room. In trauma-related settings, it accounts for approximately 30-40% of all deaths, particularly those occurring within the first 24 hours of injury [3]. Perioperatively, occult hypovolemia is not uncommon, especially in patients with sepsis, gastrointestinal bleeding, trauma, third-spacing, or other dehydration-related abnormalities [2,4]. Anesthesiologists must remain vigilant for subtle signs of intravascular depletion, as the induction of anesthesia can exacerbate vasodilation and venous pooling, precipitating sudden cardiovascular collapse in vulnerable patients.

The pathophysiology of hypovolemic shock involves complex compensatory mechanisms. In the early stages, the sympathetic nervous system triggers peripheral vasoconstriction and tachycardia to preserve blood pressure and maintain perfusion to core organs [5]. However, as volume loss continues, these compensations become insufficient, resulting in hypotension, impaired oxygen delivery, metabolic acidosis, and ultimately, cardiac arrest if untreated [5, 6]. Delayed recognition of shock is possible when relying solely on systolic blood pressure measurements, as compensatory mechanisms can prevent a noticeable drop in systolic pressure until as much as 30% of the patient’s blood volume is lost [3]. This highlights the importance of early recognition and intervention.

Pulse oximetry is an essential, non-invasive tool widely used in anesthesia to monitor arterial oxygen saturation (SpO₂). The device works on the principle of differential light absorption, emitting red and infrared light through a vascular bed, typically a fingertip or earlobe, and calculating oxygen saturation based on the pulsatile changes in absorbance associated with arterial blood flow [6]. While pulse oximetry provides real-time insights into oxygenation, it does not directly measure perfusion or volume status. Nonetheless, the quality of the oximeter waveform can be indirectly influenced by hemodynamic status, especially in hypovolemia.

Notably, several studies have demonstrated that hypovolemia can significantly alter pulse oximeter waveform characteristics. Decreased stroke volume and peripheral perfusion can lead to weak or absent plethysmographic signals, which may manifest clinically as erratic or unreadable SpO₂ values despite adequate oxygenation [7]. Such signal abnormalities are particularly important in the perioperative period, where patients may be sedated or anesthetized and unable to report symptoms of hypoperfusion. While pulse oximetry has known limitations, such as vulnerability to motion artifacts, difficulty transmitting signals in low-perfusion conditions, and interference from nail polish or cold extremities, it should not be overlooked as an indirect indicator of hemodynamic compromise [6].

This case report highlights the potential utility of an unreliable pulse oximetry signal in multiple sites as an early clinical clue of severe hypovolemia in the perioperative setting, focusing on how an instance of delayed recognition may have contributed to cardiac arrest and the need for cardiopulmonary resuscitation (CPR) during anesthetic induction.

## Case presentation

A 68-year-old man with a history of hypertension, chronic back pain, and a 20-pack-year smoking history presented with a three-day history of abdominal pain. On arrival to the emergency room, he was hypotensive, hypoxic, tachycardic with a heart rate of 110 beats per minute, and confused. He was subsequently diagnosed with a perforated duodenal ulcer with sepsis. Notably, the patient had a poor pulse oximetry signal preoperatively, despite receiving 1 L of normal saline and 2 L of lactated Ringer’s solution, which could have been an early sign of his severe hypovolemia. Attempts were made to obtain a reading by repositioning the probe from the ear to the finger and then to the forehead without success.

The patient’s perioperative course was complicated by a cardiac arrest during anesthesia induction, requiring two minutes of CPR and one round of epinephrine before achieving a return of spontaneous circulation. He received a total of 2 mg of intravenous epinephrine, 1 L of 5% albumin, and a norepinephrine infusion in addition to ongoing fluid resuscitation, with a central venous line, which stabilized his hemodynamics. Induction agents included propofol (150 mg) and fentanyl (100 mcg).

He underwent an exploratory laparotomy with pyloric exclusion, primary repair of the duodenal ulcer with an omental buttress, and gastrojejunostomy. Postoperatively, the patient recovered well, was extubated, and was weaned from vasopressors by postoperative day 1. He was transferred to a step-down unit three days later.

## Discussion

The patient presented with a perforated duodenal ulcer, hypotension, and sepsis, a critical combination associated with high morbidity and mortality. A perforated duodenal ulcer is a life-threatening condition, associated with a mortality rate of nearly 30%, largely due to complications such as peritonitis and sepsis [8]. The ulcer penetrates the intestinal wall, allowing duodenal contents to leak into the peritoneal cavity, triggering a systemic inflammatory response and potential multiorgan failure [2,5,9]. Sepsis is responsible for approximately half of all deaths in patients with perforated duodenal ulcers [8]. One of the most reliable diagnostic tools for this condition is a double-contrast CT scan, and definitive management typically involves emergent surgery to reduce mortality risk [9]. The surgical procedure usually involves peritoneal lavage with 5-10 L of saline, interrupted sutured closure of the perforated ulcer, and placement of an omental patch [10]. Additionally, a surgical drain is often inserted to remove residual fluid and is typically removed after three-five days [10]. In some cases, surgery may be avoided in patients under 70 years old who present within the first 24 hours of onset and are hemodynamically stable, with close monitoring used as the alternative approach [11].

A pulse oximeter is a noninvasive device that measures arterial blood oxygen saturation, SpO_2_ [6]. It functions by detecting changes in the absorption of red and infrared light during the cardiac cycle, reflecting the variable volume of arterial blood, as venous and capillary volumes generally remain constant [6]. However, the device does not provide direct information on blood pressure or volume status [6]. Notably, one study identified a strong correlation between decreased stroke volume and reductions in pulse amplitude, width, and area [7]. As a result, inconsistencies in SpO_2_ readings may be due to low blood volume, which limits the ability of the oximeter to record stable measurements. Once intravascular volume is restored, pulse oximeter waveform features typically return to baseline levels [7]. In this patient, inconsistent SpO_2_ measurements in combination with hypotension strongly pointed toward the possibility of hypovolemia.

In early hypovolemia, compensatory mechanisms, such as sympathetic activation, maintain vital organ perfusion through vasoconstriction and tachycardia [12]. If untreated, hypovolemia can progress to cardiovascular collapse and death [12]. An optimal resuscitation strategy aims to restore adequate stroke volume to improve tissue perfusion and oxygen delivery, while also preventing hypervolemia that can lead to venous congestion and interstitial edema [5]. In this case, early fluid resuscitation may have prevented the need for CPR. Fluid resuscitation typically involves administering 30 mL/kg of crystalloid within the first three hours [13]. Colloids are an alternative, though they have not demonstrated significant short-term survival benefits over crystalloids [14]. A key goal of fluid therapy is the restoration of adequate perfusion [12]. Clinicians are gradually shifting their focus from defining hypovolemic shock by a fixed percentage of blood loss to monitoring the patient’s response to initial fluid resuscitation [12]. 

In this case, the patient experienced cardiac arrest during anesthesia induction, a period particularly vulnerable to decompensation in hypovolemic patients due to vasodilatory effects. The repeated failure to obtain consistent SpO₂ readings preoperatively could have served as an early warning sign. Despite not being a definitive measure of hypovolemia, repeated abnormal or absent pulse oximetry signals, especially in hypotensive patients, should raise concern for hemodynamic instability and initiate early resuscitative efforts.

## Conclusions

This case highlights the clinical relevance of inconsistent pulse oximeter readings across multiple sites as a potential early sign of hypovolemia. Overlooking this subtle but critical indicator contributed to the patient’s progression to cardiac arrest and the need for CPR, which might have been avoided with earlier fluid resuscitation. While pulse oximetry is not a direct measure of volume status, poor signal quality, especially across multiple sites, should prompt urgent evaluation for hypovolemia. Clinicians should be vigilant for atypical oximetry findings in hemodynamically vulnerable patients.
